# Causal Association of Obesity and Dyslipidemia with Type 2 Diabetes: A Two-Sample Mendelian Randomization Study

**DOI:** 10.3390/genes13122407

**Published:** 2022-12-19

**Authors:** Young Lee, Ye An Kim, Je Hyun Seo

**Affiliations:** 1 Veterans Medical Research Institute, Veterans Health Service Medical Center, Seoul 05368, Republic of Korea; 2 Department of Applied Statistics, Chung-Ang University, Seoul 06974, Republic of Korea; 3 Division of Endocrinology, Department of Internal Medicine, Veterans Health Service Medical Center, Seoul 05368, Republic of Korea

**Keywords:** dyslipidemia, obesity, type 2 diabetes, single-nucleotide polymorphisms, Mendelian randomization

## Abstract

Recent studies have suggested an association between obesity and dyslipidemia in the development of type 2 diabetes (T2D). The purpose of this study was to explore the causal effects of obesity and dyslipidemia on T2D risk in Asians. Two-sample Mendelian randomization (MR) analyses were performed to assess genetically predicted obesity using body mass index (BMI) and dyslipidemia using high-density lipoprotein cholesterol (HDL), low-density lipoprotein cholesterol (LDL), total cholesterol (TCHL), and triglycerides (TG) versus T2D susceptibility using genome-wide association study (GWAS) results derived from the summary statistics of Biobank Japan (*n* = 179,000) and DIAbetes Meta-ANalysis of Trans-Ethnic association studies (*n* = 50,533). The MR analysis demonstrated evidence of a causal effect of higher BMI on the risk of T2D (odds ratio (OR) > 1.0, *p* < 0.05). In addition, TG showed a protective effect on the risk of T2D (ORs 0.68–0.85). However, HDL, LDL, and TCHL showed little genetic evidence supporting a causal association between dyslipidemia and T2D. We found strong genetic evidence supporting a causal association of BMI with T2D. Although HDL, LDL, and TCHL did not show a causal association with T2D, TG had a causal relationship with the decrease of T2D. Although it was predicted that TG would be linked to a higher risk of T2D, it actually exhibited a paradoxical protective effect against T2D, which requires further investigation.

## 1. Introduction

Type 2 diabetes (T2D) is a complex metabolic disease characterized by hyperglycemia due to defects in insulin secretion, action, or both [1]. Globally, T2D is a prevalent public health issue, affecting 422 million adults (in 2016) and resulting in 1.6 million deaths [2]. The number of diabetic individuals is predicted to reach 300 million by 2025 [3]. Even more serious, long-term diabetes can lead to microvascular complications, such as diabetic kidney disease and diabetic retinopathy [4,5,6,7,8,9]. Consequently, early detection and management of T2D risk factors are critical for disease prevention.

Insulin resistance, one of the pathophysiological characteristics of metabolic syndrome (MetS) and T2D, is frequently related with obesity and dyslipidemia. Obesity, as measured by body mass index (BMI), is a well-known risk factor for T2D [10]. Additionally, the relationship of dyslipidemia with T2D risk has been supported by increasing evidence in recent years; the use of lipid-lowering medications, such as statin therapy, has slightly increased the risk of T2D development [11]. Observation studies have shown that prediabetic and diabetic states are frequently associated with high triglyceride (TG) and low high-density lipoprotein cholesterol (HDL) levels [12,13]. Although pre-existing T2D often increases low-density lipoprotein cholesterol (LDL) level and lowers HDL, previous investigations have demonstrated that elevated LDL cholesterol level hinders glucose tolerance, and that a high ratio of total cholesterol (TCHL) to HDL is predictive of T2D [14,15,16]. Additionally, TG and TG/HDL are independent risk factors for T2D, with TG/HDL being a greater risk factor than TG [17]. In addition, high TCHL showed an increased hazard ratio (1.139, 95% confidence interval (CI): 1.116 to 1.163) in data from 2,827,950 Koreans [18]. These results of association studies from real-world data suggest that a causal association study is necessary.

Mendelian randomization (MR) is a genetic epidemiology technique that uses genetic variants associated with potential exposures as instrumental variables (IVs) to assess their causal effects on disease outcomes [19,20]. Using this methodology, several studies have examined the causality of risk factors identified in observational studies of T2D [21,22]. While BMI was associated with high T2D (odds ratio (OR) = 1.27, 95% CI: 1.18 to 1.36), the effects of dyslipidemia on T2D were controversial [21]. A recent study using wide-angle MR confirmed that adult BMI was causally associated with T2D (OR = 1.89, 95% CI: 1.73 to 2.07) and HDL showed causality for T2D (OR = 0.78, 95% CI: 0.67 to 0.91) [23]. Since the majority of studies have been conducted on Europeans, there is a need for analytical research using the recently disclosed Asian genetic data to test the MR hypothesis. To achieve this goal, we conducted two-sample MR using multi-cohort summary statistics of an Asian population for BMI, TG, HDL, LDL, and TCHL levels.

## 2. Materials and Methods

### 2.1. Study Design Overview

**Figure 1:**
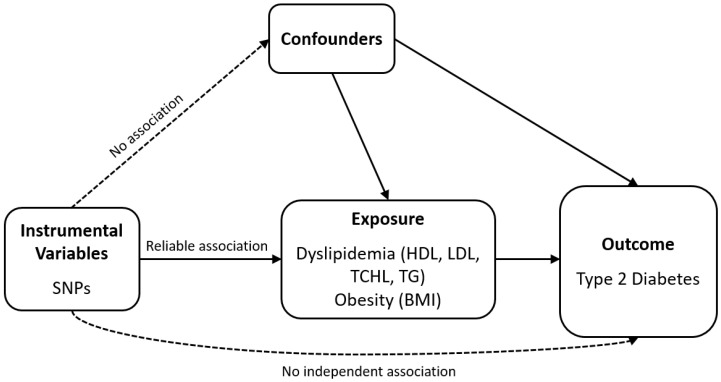
Schematic of the analytical study design. SNP: single-nucleotide polymorphism; HDL: high-density lipoprotein cholesterol; LDL: low-density lipoprotein cholesterol; TCHL: total cholesterol; TG: triglycerides; BMI: body mass index.

Schematic plots of the analytical study design are shown in Figure 1. The Institutional Review Boards of the Veterans Health Service Medical Center approved this study protocol and waived the need for informed consent (IRB No. 2019-01-012 and IRB No. 2022-11-004) since this study was performed in a retrospective manner in compliance with the Helsinki Declaration.

### 2.2. Data Source

The datasets for the genome-wide association studies (GWASs) summary statistics used in this analysis were from the GWAS Catalog (available at https://www.ebi.ac.uk/gwas/summary-statistics, accessed on 19 July 2022). GWAS data on BMI and dyslipidemia were adopted from Biobank Japan (BBJ, *n* = 179,000) for the East Asian population [24], while T2D GWAS data from 50,533 samples (16,677 cases and 33,856 controls) were collected from a DIAbetes Meta-ANalysis of Trans-Ethnic association studies (DIAMANTE) project for a South Asian population [25]. The datasets for summary statistics are described in detail in Table 1.

Single-nucleotide polymorphisms (SNPs) associated with exposure at the genome-wide significance threshold (*p* < 5.0 × 10^−8^) were used as IVs. To ensure that each IV was independent of the others, we pruned theses SNPs by linkage disequilibrium (LD; *r^2^* = 0.001, clumping distance = 10,000 kb). The 1000 Genome Phase III East Asian data were used as a reference to calculate linkage disequilibrium between the SNPs. The *F* statistics were computed to detect weak instruments problems. *F* statistics greater than 10 are considered to present no evidence of weak instrument bias [26].

### 2.3. Mendelian Randomization

MR analysis was performed with the following assumptions: (1) genetic variants should have substantial association with exposure; (2) these variants should not be related to confounders of the exposure–outcome relationship; and (3) these variants should only affect the outcome through the exposure (i.e., no directional horizontal pleiotropy effect).

We used inverse variance-weighted (IVW) MR with multiplicative random effects as our primary analysis method [26,27]. Additional methods included the weighted median [28], MR-Egger regression (with or without adjustment via Simulation Extrapolation (SIMEX) method) [29,30], and MR polyhedral sum of residuals and outliers (MR-PRESSO) [31]. When all genetic variations meet the three IV assumptions, IVW is the approach with the highest efficiency [32]. While the estimate of IVW might be biased when one or more of the variants are invalid [28], the weighted median method produces precise estimates of causality even when as many as 50% of the instruments are incorrect [28]. The MR-Egger method enables the estimation of suitable causal effects even in the presence of pleiotropic effects, allowing a non-zero intercept that represents the average horizontal pleiotropic effects [29]. The MR-Egger with SIMEX can be used to correct bias when the no measurement error (NOME) assumption is not met (*I*^2^ value < 90%) [30]. The MR-PRESSO test, which identifies outliers, corrects the IVW analysis results for horizontal pleiotropy by deleting outliers [31].

Heterogeneity for IVW and MR-Egger was evaluated using the Cochran’s Q statistic and the Rücker’s Q′ statistic, respectively [27,33]. The heterogeneity suggested that the genetic variations might be pleiotropic [27,34]. Additionally, directional horizontal pleiotropy was assessed by the MR-PRESSO global test. A *p* value less than 0.05 for Cochran’s Q statistic, Rücker’s Q′ statistic, or the MR-PRESSO global test indicated possible pleiotropy in the genetic variations. The significance level for analyses in this study was 0.05. All analyses were performed using the TwoSampleMR and simex packages in R version 3.6.3 (R Core Team, Vienna, Austria).

## 3. Results

### 3.1. Genetic Instrumental Variables

Numbers of IVs were 79, 48, 31, 47, and 37 for BMI, HDL, LDL, TCHL, and TG, respectively. The *F* statistics for BMI (from 30.47 to 384.02), HDL (from 30.31 to 1216.64), LDL (from 30.35 to 1246.70), TCHL (from 30.50 to 931.94), and TG (from 31.22 to 1403.81) used for MR were greater than 10, demonstrating a small chance of weak instrument bias. Detailed information of the IVs used in this study is provided in Supplementary Table S1. Cochran’s Q test revealed that the IVs were heterogeneous (Table 2), so the random-effect IVW approach was employed. Rücker’s Q′ test from MR-Egger also revealed heterogeneity between IVs; however, the MR–Egger regression intercepts showed no horizontal pleiotropic effect (all *P*s > 0.05) regardless of SIMEX adjustment (Table 2). The MR–PRESSO global test (Table 2) demonstrated horizontal pleiotropy in all IVW analyses (all *P*s < 0.05). Outlier IVs with a horizontal pleiotropic effect are indicated in Supplementary Table S1.

### 3.2. Mendelian 

#### Randomization for Obesity (BMI)

**Figure 2:**
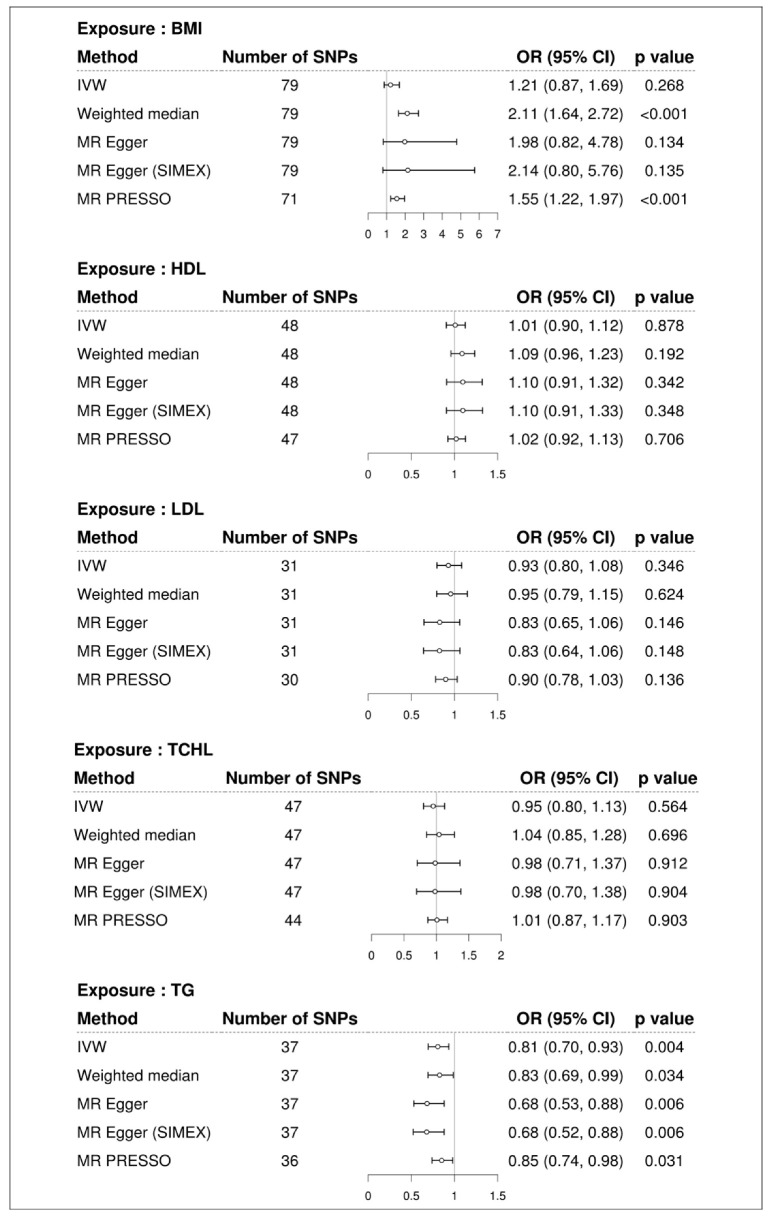
Forest plot of causal associations of BMI and dyslipidemia with T2D. BMI: body mass index; T2D: type 2 diabetes; SNP: single nucleotide polymorphism; OR: odds ratio; CI: confidence interval; IVW: inverse-variance weight; MR: Mendelian randomization; SIMEX: simulation extrapolation; PRESSO: polyhedral sum of residuals and outliers; HDL: high-density lipoprotein cholesterol; LDL: low-density lipoprotein cholesterol; TCHL: total cholesterol; TG: triglycerides.

**Figure 3:**
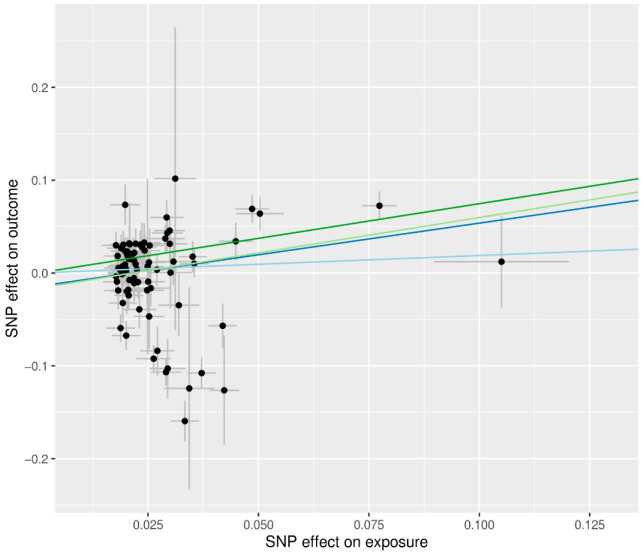
Scatter plots of MR tests assessing the effect of BMI on T2D. The dots represent the effect size (β) of each SNP on BMI (*x*-axis) and T2D (*y*-axis), and the grey lines show their standard errors. Regression slopes show the estimated causal effect of BMI on T2D. The light blue, dark blue, light green, and dark green regression lines represent the IVW, MR-Egger, MR-Egger (SIMEX), and weighted median estimate, respectively. MR: Mendelian randomization; BMI: body mass index; T2D: type 2 diabetes; SNP: single nucleotide polymorphism; IVW: inverse-variance weight; SIMEX: simulation extrapolation.

Higher BMI was associated with increased risk of T2D in all MR methods (ORs > 1), but statistical significance depended on the method (Figure 2). Even in the presence of pleiotropic effects, the MR-Egger method can adequately estimate causal effects; however, this method has less power than other approaches. Since there was no significant evidence of pleiotropy (MR-Egger intercept −0.014, standard error 0.012, *p* = 0.241, Table 2), IVW is preferred over MR-Egger. We advise examining the MR-PRESSO result, which is calculated using IVW, after removing outliers because the MR-PRESSO global test for BMI was significant (*p* < 0.001, Table 2). In MR PRESSO, higher BMI was significantly associated with increased risk of T2D (MR-PRESSO OR = 1.55, 95% CI: 1.22 to 1.97, *p* < 0.001, eight SNPs excluded), which is consistent with the weighted median (weighted median MR OR = 2.11, 95% CI: 1.64 to 2.72, *p* < 0.001). A scatter plot showed genetic associations of BMI against genetic associations with T2D for each SNP (Figure 3; light blue, dark blue, light green, and dark green regression lines represent the IVW, MR-Egger, MR-Egger (SIMEX), and weighted median estimate, respectively).

### 3.3. Mendelian Randomization for Dyslipidemia (HDL, LDL, TCHL, TG)

**Figure 4:**
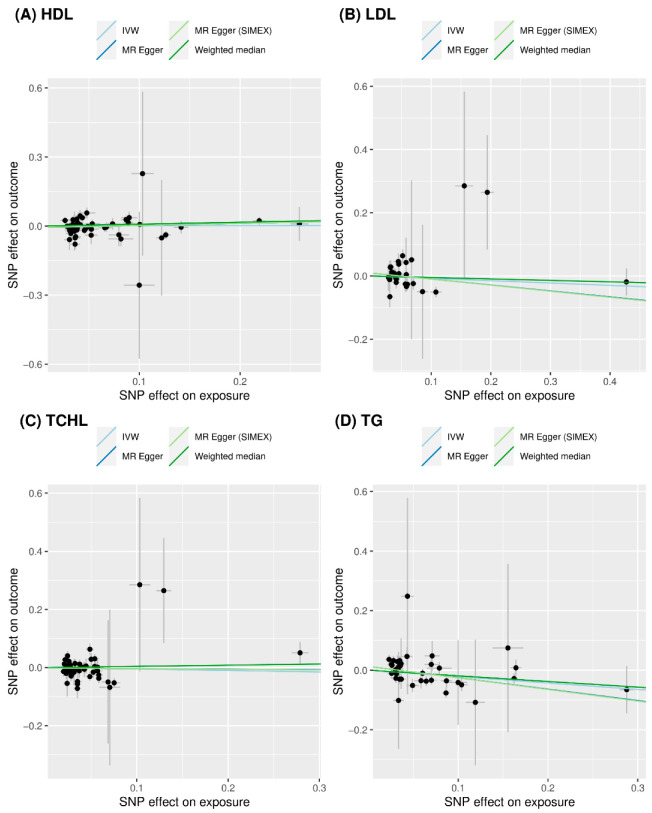
Scatter plots of MR tests assessing the effect of dyslipidemia on T2D. (**A**) Causal effect of HDL on T2D; (**B**) causal effect of LDL on T2D; (**C**) causal effect of TCHL on T2D; and (**D**) causal effect of TG on T2D. The dots represent the effect sizes (β) of each SNP on dyslipidemia (*x*-axis) and T2D (*y*-axis), and the grey lines show their standard errors. Regression slopes show the estimated causal effect of dyslipidemia on T2D. The light blue, dark blue, light green, and dark green regression lines represent the IVW, MR-Egger, MR-Egger (SIMEX), and weighted median estimate, respectively. MR: Mendelian randomization; T2D: type 2 diabetes; HDL: high-density lipoprotein cholesterol; LDL: low-density lipoprotein cholesterol; TCHL: total cholesterol; TG: triglycerides; SNP: single nucleotide polymorphism; IVW: inverse-variance weight; SIMEX: simulation extrapolation.

We did not find evidence supporting a causal association between HDL and risk of T2D (IVW MR OR = 1.01, 95% CI: 0.90 to 1.12, *p* = 0.878; MR-PRESSO OR = 1.02, 95% CI: 0.92 to 1.13, *p* = 0.706, one SNP excluded), between LDL and risk of T2D (IVW MR OR = 0.93, 95% CI: 0.80 to 1.08, *p* = 0.346; MR-PRESSO OR = 0.90, 95% CI: 0.78 to 1.03, *p* = 0.136, one SNP excluded), and between TCHL and risk of T2D (IVW MR OR = 0.95, 95% CI: 0.80 to 1.13, *p* = 0.564; MR-PRESSO OR = 1.01, 95% CI: 0.87 to 1.17, *p* = 0.903, three SNPs excluded) using the IVW approach. Similar results were obtained using the weighted median and MR-Egger and the MR-Egger (SIMEX). All MR methods showed that TG significantly reduced the risk of T2D (IVW MR OR = 0.81, 95% CI: 0.70 to 0.93, *p* = 0.004; weighted median MR OR = 0.83, 95% CI: 0.69 to 0.99, *p* = 0.034; MR-Egger OR = 0.68, 95% CI: 0.53 to 0.88, *p* = 0.006; MR-Egger (SIMEX) OR = 0.68, 95% CI: 0.52 to 0.88, *p* = 0.006; MR-PRESSO OR = 0.85, 95% CI: 0.74 to 0.98, *p* = 0.031, one SNP excluded). Scatter plots (Figure 4) also demonstrated a causal relationship between higher TG and reduced risk of T2D, but not with HDL, LDL, and TCHL.

## 4. Discussion

In this study, we demonstrated positive causal evidence that higher BMI genetic susceptibility increases the risk of T2D. In addition, genetic evidence of a high TG protective effect on T2D was demonstrated. However, LDL, HDL, and TCHL showed little genetic evidence to support a causal association between dyslipidemia and T2D.

Our study found that higher BMI is a causal risk factor for T2D; these findings are consistent with those of previous conventional observational studies [35,36,37] and MR studies [38,39,40]. The link between obesity and higher risk of T2D is well established and supported by several studies [10,41,42]. According to a meta-analysis, the chance of developing diabetes increased by 1.18 (95% CI: 1.16 to 1.20) per unit of BMI [35]. In addition, a meta-analysis using 27 cohorts of 154,989 individuals from the Asia-Pacific region found a positive correlation between BMI and risk of T2D, with each 2 kg/m^2^ decrease in BMI associated with a 27% (95% CI: 23 to 30) decreased risk of T2D [36]. Another meta-analysis using prospective cohort studies revealed that the relative risk of T2D for obesity was 7.28 (95% CI: 6.47 to 8.28), and the relative risk for overweight individuals was 2.92 (95% CI: 2.57 to 3.32) compared to individuals with normal weight [37]. According to MR studies with subjects of European ancestry, higher BMI had a causal relationship with increased risk of T2D (OR = 1.98, 95% CI: 1.41 to 2.78 [38] and OR = 2.74, 95% CI: 2.42 to 3.10 [39] per one standard deviation). In another MR study with three Chinese Han cohorts (*n* = 6,476), there was no significant relationship between BMI and glucose deterioration (β = 0.922, 95% CI: 0.83 to 1.02), but there was a causal relationship between higher waist to hip ratio and glucose deterioration (β = 1.66, 95% CI: 1.07 to 2.57) [40]. The effect of BMI on T2D in the Chinese study was the same as in our study, but the effect was not statistically significant. Because of the smaller sample size and different set of SNPs as instrumental variables, the findings of the Han Chinese cohort study differ from those of our study.

Several epidemiological studies have a demonstrated positive correlation between TG level and risk of T2D [14,43,44,45,46]. We hypothesized a causal association of TG and T2D, since several studies have indicated a positive connection between high TG level and T2D. In our MR analysis, however, high TG level was associated with a lower risk of T2D. A previous genetic study has shown that TG-increasing alleles are associated with protection against T2D [47]. According to a MR study with patients of European ancestry, higher TG was associated with a lower risk of T2D (OR = 0.83, 95% CI: 0.72 to 0.95 from MR-Egger) [48], consistent with our findings. Despite the lack of association between TG and risk of T2D in the IVW method (OR 1.01, 95% CI: 0.91 to 1.11), they suggest that TG may play a protective role in T2D [48]. Because other MR investigations [23,49] have demonstrated that TG is not related to risk of T2D, our finding that TG lowers the risk of T2D is controversial. To address these discrepancies, additional research is required.

The true strength of this study includes the utilization of relatively large Asian cohort data that provides causal association of obesity and dyslipidemia on T2D. However, there are a few limitations of this study. First, we were unable to explain various confounding factors in this study based on two-sample MR using summary statistics because we did not have access to individual-level data. Second, since our study was entirely based on data from Asians, our findings might not generalize to other ethnic groups. Third, test methods exist to check the MR assumptions, but these methods do not guarantee complete satisfaction. As violation of MR assumptions can lead to invalid conclusions, the results should be interpreted with care.

## 5. Conclusions

We found strong genetic evidence supporting a causal association of BMI with T2D. Additionally, elevated TG level had protective effects on T2D. However, there was limited causal evidence for a link between T2D and LDL, HDL, and TCHL. We anticipated that TG would be associated with an increased risk of T2D, but we demonstrated the opposite, a paradoxical protective effect against T2D, which warrants further investigation.

## Figures and Tables

**Table 1:** Summary statistics of data.

Traits	Data Source	No. of Participants	No. of Variants	Population
BMI	BBJ Project [24]	163,835	13,479,178	East Asian
HDL		74,970	13,465,896	
LDL		72,866	13,461,863	
TCHL		135,808	13,476,599	
TG		111,667	13,471,903	
T2D	DIAMANTE project [25]	50,533 (16,677 cases + 33,856 control)	18,881,775	South Asian

HDL: high-density lipoprotein cholesterol; LDL: low-density lipoprotein cholesterol; TCHL: total cholesterol; TG: triglycerides; BMI: body mass index; T2D: type 2 diabetes; BBJ: BioBank Japan; DIAMANTE: DIAbetes Meta-ANalysis of Trans-Ethnic association studies.2.3. Selection of genetic instrumental variables.

**Table 2:** Heterogeneity and horizontal pleiotropy of instrumental variables.

Exposure					Heterogeneity		Horizontal Pleiotropy			
				Cochran’s Q Test from IVW	Rucker’s Q′ Test from MR-Egger	MR-PRESSO Global Test	MR-Egger		MR-Egger (SIMEX)	
	N	*F*	*I*^2^ (%)	*p*-Value	*p*-Value	*p*-Value	Intercept, β (SE)	*p*-Value	Intercept, β (SE)	*p*-Value
BMI	79	56.00	95.72	<0.001	<0.001	<0.001	−0.014 (0.012)	0.241	−0.016 (0.013)	0.226
HDL	48	147.86	99.01	<0.001	0.001	0.002	−0.007 (0.006)	0.291	−0.007 (0.006)	0.294
LDL	31	113.25	98.69	0.018	0.022	0.019	0.009 (0.008)	0.258	0.009 (0.008)	0.258
TCHL	47	101.50	98.22	<0.001	<0.001	<0.001	−0.002 (0.007)	0.828	−0.002 (0.008)	0.841
TG	37	178.44	99.28	<0.001	0.002	0.001	0.013 (0.008)	0.127	0.013 (0.008)	0.124

N: number of instruments; *F*: mean *F* statistic; IVW: inverse-variance weight; MR: Mendelian randomization; PRESSO: polyhedral sum of residuals and outliers; SIMEX: simulation extrapolation; β: β coefficient; SE: standard error; HDL: high-density lipoprotein cholesterol; LDL: low-density lipoprotein cholesterol; TCHL: total cholesterol; TG: triglycerides; BMI: body mass index.

## Data Availability

The datasets for the genome-wide association study (GWAS) summary statistics can be found in the GWAS Catalog (https://www.ebi.ac.uk/gwas/summary-statistics, accessed on 19 July 2022).

## Supplementary Materials

The following supporting information can be downloaded at https://www.mdpi.com/article/10.3390/genes13122407/s1: Table S1: List of single-nucleotide polymorphisms used in this MR study as instrumental variables.
